# Assets of the non-pathogenic microorganism *Dictyostelium discoideum *as a model for the study of eukaryotic extracellular vesicles

**DOI:** 10.12688/f1000research.2-73.v1

**Published:** 2013-03-04

**Authors:** Irène Tatischeff

**Affiliations:** 1 Laboratoire Jean Perrin, UPMC University of Paris 06, Paris, 75005, France; 2 Laboratoire Jean Perrin, CNRS, Paris, 75005, France

## Abstract

*Dictyostelium discoideum *microvesicles have recently been presented as a valuable model for eukaryotic extracellular vesicles. Here, the advantages of *D. discoideum *for unraveling important biological functions of extracellular vesicles in general are detailed. **** *D. discoideum,* a **non-pathogenic eukaryotic microorganism, belongs to a billion-year-old *Amoeboza *lineage, which diverged from the animal-fungal lineage after the plant animal-split. During growth and early starvation-induced development, it presents analogies with lymphocytes and macrophages with regard to motility and phagocytosis capability, respectively. Its 6-chromosome genome codes for about 12,500 genes, some showing analogies with human genes. The presence of extracellular vesicles during cell growth has been evidenced as a detoxification mechanism of various structurally unrelated drugs. Controls led to the discovery of constitutive extracellular vesicle secretion in this microorganism, which was an important point. ****It means that the secretion of extracellular vesicles occurs, in the absence of any drug, during both cell growth and early development. This constitutive secretion of *D. discoideum* cells is very likely to play a role in intercellular communication. The detoxifying secreted vesicles, which can transport drugs outside the cells, can also act as "Trojan horses", capable of transferring these drugs not only into naïve *D. discoideum *cells, but into **human cells as well. Therefore, these extracellular vesicles were proposed as a new biological drug delivery tool. ****Moreover, **** *Dictyostelium*, chosen by the NIH (USA) as a new model organism for biomedical research, has already been used for studying some human diseases. These cells, which are much easier to manipulate than human cells, can be easily designed in simple conditioned medium experiments. Owing to the increasing consensus that extracellular vesicles are probably important mediators of intercellular communication, *D. discoideum* is here suggested to constitute a convenient model for tracking as yet unknown biological functions of eukaryotic extracellular vesicles.

## Introduction

### A short four-decade story of extracellular vesicles

Four decades ago, the extracellular medium was considered to be no more than a waste reservoir of cell life, exclusively occurring inside the eukaryotic cell delimited by the plasma membrane.

Here are a few landmarks of the story of extracellular vesicles (EVs): "Exosomes" were first mentioned by R. Johnstone in 1970 and further, thoroughly studied during maturation of reticulocytes ^1, 2^. "Shed" vesicles were observed in 1981 ^3^. Exosomes in blood cells and their function in the transfer of immunity were thoroughly studied in the early 2000s ^4, 5^. Clinical observations of exosomes, as normal and pathogenic biomarkers in urine, were first mentioned in 2004 ^6^. The presence of nucleic acids, mRNAs and microRNAs in exosomes, was pointed out in 2007 ^7^, as a first strong indication of vesicle-mediated intercellular communication.

The increasing importance of EVs can be evaluated by international meetings devoted first to exosomes in Montreal, Canada in 2005 ^8^, then in 2011, in the International Workshop on Exosomes in Paris, France ^9^, and more recently, in 2012, to EVs in general, at the first meeting of the International Society of Extracellular Vesicles (ISEV) in Gothenburg, Sweden ^10^. An increasing number of workshops are currently devoted to this exploding field of research.

### Why do cells expel extracellular vesicles?

In the absence of a consensus on the nomenclature for various kinds of extracellular vesicles, the term "extracellular vesicles" is used throughout this paper to designate secreted vesicles in general, with no qualification based on defined criteria. For well known subtypes, like exosomes, the more precise qualification is mentioned, when appropriate.

After a widespread observation of many different kinds of extracellular vesicles, emerging from quite different fields of biology and medicine, the modern puzzling questions deal about the life functions of these vesicles.

Why do so many cells expand their action field beyond the cell membrane? Why do cells release vesicles? ^11^. What are the physiological functions of all these EVs? The current state of the art about the role of extracellular vesicles has recently been presented ^12^, whereas exosomes seem to be appealing candidates for mediating intercellular communication ^13^.

Human complexity is indeed much too high to suggest one particular human cell type as a model for understanding the respective roles of the "inside/outside" parts of the cell. To address such a fundamental question requires a convenient well-known eukaryotic model, easy to handle and to follow in a variety of extracellular environments.

## A model for the study of eukaryotic extracellular vesicles: *Dictyostelium discoideum*

### About *Dictyostelium discoideum*

*Dictyostelium discoideum* came to scientific life in 1935, when it was discovered by Raper ^14^ in the USA Virginia forest, but belongs to a billion year-old *Amoeboza* lineage, which diverged from the animal-fungal lineage after the plant animal-split ^15^. To survive for such a long time as a unicellular protist, it had to develop a well-conserved life strategy. One of its original biological tricks was to fight against starvation through the first known eukaryotic tentative step towards multicellularity, that is, the aggregation of undifferentiated cells. This aggregation is followed by differentiation into two main cell species: dying stalk cells and "everlasting" spores. Thus, *D. discoideum* experiences specific time-separated physiological states of growth and differentiation during its life cycle. During growth, as unicellular non-pathogenic amoebae, *D. discoideum* cells, about 10 µm in diameter, are very similar to some human blood cells; they are often compared to lymphocytes with regard to motility, and to macrophages concerning their capability for phagocytosis. After about 24h of starvation-induced development, through cell aggregation (of about 10 ^5^ cells), followed by differentiation and morphogenesis, it ends up as a "mushroom-like" organism of 1–2 mm height, forming a fruiting body, composed of a stalk (about 20% of the cells) and a spore bag containing viable spores (about 80% of the cells) ^16^.

Thus, whereas differentiation pulls the microorganism towards a plant-like structure, growing and aggregating cells remain as animal-like cells. Therefore, *D. discoideum* can offer a simple model of individual eukaryotic cells during growth and also mimic an undifferentiated "tissue" during starvation-induced aggregation. The above-defined "animal" state of *D. discoideum* during growth and aggregation, completely separated from differentiation, is the first asset of *D. discoideum* as a model for eukaryotic cells. Some molecular approaches to cell biology using *D. discoideum* are detailed in *Methods in Cell Biology* ^17^.

Another advantage of *D. discoideum* is the good knowledge of its genetic material ^18^. Its 6-chromosome genome has been completely sequenced ^15^. The relatively small genomic DNA (3.4 × 10 ^7^ bp) is efficiently transcribed (90%) into about 12,500 genes, some of which show analogies with human genes. Its 55.5 kb mitochondrial genome has also been sequenced ^19^. A multicopy 90 kb extrachromosomal element that carries the ribosomal RNA genes, and some plasmids complete the DNA components. The RNA amount is about 10 times the DNA amount and due to this RNA excess, the cell cycle does not require a G1 RNA-synthesis step before the DNA-synthesis S phase. Moreover, *Dictyostelium* cells can be easily transformed by restriction enzyme-mediated integration (REMI) ^20^.

A third asset of *D. discoideum* lies in its simple growth conditions. In nature, *D. discoideum* cells grow by bacterial phagocytosis, with a 3h generation time. A widely used laboratory double axenic mutant, Ax-2, can grow in semi-synthetic HL5-medium ^21^, either as adhering cells or in agitated suspension, with a 8 - 11h generation time. A completely defined medium has also been elaborated ^22^ and is now commercialised, as are many other *Dictyostelium* growth media (Foremedium, UK). Moreover, *D. discoideum* cells can reach a density of 2–3 × 10 ^7^ cells ml ^-1^ in the stationary phase and can also be grown in bioreactors ^23^, when huge amounts of cell materials are needed.

It is worth noting that, owing to all these advantages, *D. discoideum* was recognised, in 1999, by the National Institutes of Health (NIH, USA) as a new interesting model for biomedical research. Indeed, *Dictyostelium* had already helped in the understanding of the function of nucleoside diphosphate kinase (NDP) involved in tumour metastasis ^24, 25^, and in elucidating the structure of vaults ^26, 27^ linked to the drug resistance-related protein LRP ^28^. More recently, R. Escalante gathered various studies that have used *D. discoideum* as a model for human diseases ^29^. They include pathogen infections, sensitivity to cisplatin and other anticancer drugs, pathobiology of cell motility, lissencephaly, bipolar disorder treatments, lysosomal trafficking and mitochondrial diseases.

### Appearance of *D. discoideum* in the field of extracellular vesicles

**Drug detoxification in *D. discoideum* cells is mediated through release of extracellular vesicles containing the drug.** In 1998, it was shown that *Dictyostelium* cells, known to be highly resistant to xenobiotics, get rid of Hoechst 33342 (HO342), a vital DNA stain for most cells, but not for *D. discoideum* cells, by means of secretion of extracellular vesicles embedding the dye ^30^. This capability for *D. discoideum* extracellular vesicle-mediated detoxification has also been checked for hydrophobic drugs, like the photosensitizer hypericin, used in some cancer diagnoses and aimed to photodynamic therapy ^31^.

***D. discoideum* extracellular vesicles can transfer their vesicular cargos to other cells.** Previous studies have found that *D. discoideum* drug-containing extracellular vesicles are able to transfer HO342 not only to the nuclei of naïve HO342-resistant *Dictyostelium* cells, but also to the nuclei of human leukaemic multidrug-resistant K562r cells ^32^. In the same way, it it has been shown that hypericin can be transferred from hypericin-containing *Dictyostelium* extracellular vesicles to the Golgi of tumoral HeLa cells ^31^. Taking into account these experiments, *Dictyostelium* extracellular vesicles have been proposed as a new biological therapeutic drug delivery tool ^33, 34^.

***D. discoideum* extracellular vesicles are not only a cell detoxifying tool.** These vesicles are constitutively released during both growth ^30^ and starvation-induced aggregation ^35^, and are thus candidates for the mediation of intercellular communication.

Quite recently, C. A. Parent found and characterised exosomes during *Dictyostelium* aggregate formation, suggesting that these organelles are conserved in both *Dictyostelium* and mammals ^36, 37^ (personal communication).

### A model to unravel biological functions of eukaryotic extracellular vesicles

In order to use *D. discoideum* EVs as a model for the functional study of eukaryotic EVs, their characterisation is needed. Recently, these vesicles were used to propose a fast characterisation of EVs by means of three joined techniques: nanoparticle tracking analysis, cryo-electron microscopy and Raman tweezer microspectroscopy ^38^.

With regard to the precise characterisation of *Dictyostelium* EVs, much remains to be done.

First chacterisations of EVs lipids and nucleic acids ^30^ and of EVs proteins ^31^ have been performed. A more precise protocol for the purification of these extracellular vesicles should be devised, followed by a thorough characterisation of the components of all the different subclasses of *D. discoideum* extracellular vesicles.

However, before performing a specialised study of each subclass of *D. discoideum* extracellular vesicles, it is worth questioning the whole distribution panel of EVs accompanying each important physiological state of *D. discoideum* cell life. R. Gomer was a pioneer in stressing the importance of the conditioned extracellular medium of *D. discoideum* cells as a source of secreted autocrine factors during both growth and early starvation ^39, 40^. He showed that the aggregate sizes were controlled by autocrine protein factors in the extracellular medium ^41^ and showed the presence of autocrine cell growth inhibition ^42^.

Experiments are currently undertaken to test the plausible hypothesis that cells are not programmed to grow, differentiate or die by an apoptotic "clean" death, by their only genomic content and that cells would need intercellular communications to define their individual fate. The necessary signals would be conditioned by all the surrounding cells, according to their own history, and the most convenient way to communicate should indeed be searched for in the cell extracellular medium.

Previously, we found that an apoptotic-like death could be induced with full efficiency by starving a naïve *D. discoideum* cell population in a conditioned medium. This conditioned medium (t _22_) was prepared by starving a first *D. discoideum* cell population in agitated suspension in a phosphate buffer (pH 6.8) for 22 h. When starved in this cell-depleted t _22_ conditioned medium, the second *D. discoideum* cell population completely lost its aggregation competence and was directed to an apoptosis-like death ^35, 43^. Preliminary (unpublished) experiments indicated that the extracellular vesicles present in the t _22_ conditioned medium might be responsible for changing the fate of *D. discoideum* cells from "social" aggregation to mitochondrial-mediated apoptotic death.

In order to understand the important biological communication signals potentially carried by *D. discoideum* EVs, it is interesting to take advantage of the relative simplicity of this eukaryotic model. *D. discoideum* is indeed a wonderful tool to question the respective functions of and interplay between extracellular vesicles and soluble secreted autocrine factors in conditioned medium experiments. This should help in understanding the main, still mostly unknown, biological functions of the secreted vesicles.

## Conclusion

In 2012, *Dictyostelium discoideum* was recognized as an interesting model for the study of eukaryotic extracellular vesicles ^10, 44^. Due to the important knowledge accumulated on this eukaryotic microorganism since 1935, and to the wide panel of well-defined mutants available in the Stock Center (see dictyBase), a lot of simple experiments can be designed to question the physiological functions of EVs during cell growth, aggregation or apoptotic-like cell death. Moreover, the findings obtained with *D. discoideum* are likely to be extrapolated to higher eukaryotic cells, in the same way as the extracellular vesicle-mediated detoxification mechanism of *D. discoideum* cells was a clue towards the introduction of a new multidrug resistance mechanism against antitumoral chemotherapy ^45^.
