# Supplementary material for: Assets of the non-pathogenic microorganism Dictyostelium discoideum as a model for the study of eukaryotic extracellular vesicles
Source: F1000Res. 2013 Mar 4;2:73. [Version 1] doi: 10.12688/f1000research.2-73.v1 (PMC3782363; doi:10.12688/f1000research.2-73.v1)
Supplement: Peer review history

# Peer review: Referee response for version 1

**Published:** 2013-04-04

**Authors:** Lawrence Rajendran

**Affiliations:** 1 Division of Psychiatry Research, University of Zurich, Zurich, Switzerland

In the review, Dr. Tatischeff presents the advantages of using *D. discoideum* as a model system to study extracellular vesicle release. It is a very well written review and is up to date. Dr. Tatischeff has performed pioneering work in this field and has now, in this recent review, provided a comprehensive overview on the history of *D. discoideum* extracellular vesicle release research.

I have read this submission. I believe that I have an appropriate level of expertise to confirm that it is of an acceptable scientific standard.

# Peer review: Referee response for version 1

**Published:** 2013-03-06

**Authors:** Richard Gomer

**Affiliations:** 1 Department of Biology, Texas A&M University, College Station, TX, USA

This is a nice brief review of the work on *Dictyostelium* exosomes.

I have read this submission. I believe that I have an appropriate level of expertise to confirm that it is of an acceptable scientific standard.
